# Uncertainty in Estimates, Incentives, and Emission Reductions in REDD+ Projects

**DOI:** 10.3390/ijerph15071544

**Published:** 2018-07-21

**Authors:** Jichuan Sheng, Weihai Zhou, Alex de Sherbinin

**Affiliations:** 1 Institute of Climate Change and Public Policy, Nanjing University of Information Science & Technology, 219 Ningliu Road, Nanjing 210044, Jiangsu, China; 2 Business School, Nanjing University of Information Science & Technology, 219 Ningliu Road, Nanjing 210044, Jiangsu, China; zhouweihai@nuist.edu.cn; 3 Center for International Earth Science Information Network (CIESIN), The Earth Institute at Columbia University, P.O. Box 1000, Palisades, NY 10964, USA; adesherbinin@ciesin.columbia.edu

**Keywords:** REDD+, deforestation, incentive, uncertainty, performance

## Abstract

The accurate monitoring and measurement of emission reductions is a critical step in Reducing Emissions from Deforestation and Degradation (REDD+). However, the existence of uncertainty in emission reduction estimates affects the performance of REDD+ projects. We assert that incentive could be a valuable policy tool for reducing monitoring errors and transaction costs. Using Stackelberg models and simulation research, this paper examines the effects of uncertainty and incentive on performance and stakeholder benefits of REDD+ projects. Finally, the uncertainties in REDD+ projects are further discussed, and equilibrium errors, emission reductions, and stakeholder benefits in different scenarios are compared. The results show that errors do affect the measured value of carbon emissions and compensation payments. However, incentive for investors can reduce monitoring errors and improve the performance of REDD+ projects. Therefore, in the future, incentive should be provided to investors rather than landholders.

## 1. Introduction

The absorption of CO_2_ by forest land is a critical means of carbon capture and storage. Tropical forests account for only 15% of the global land area, but absorb about 25% of the carbon in the terrestrial biosphere [1]. A total of 28.9 billion tons of carbon were stored in the global forest in 2010 [2]. However, greenhouse gas emissions from deforestation and forest degradation have become the second major cause of global warming, which has accounted for 12%–20% of the total amount of carbon emissions caused by human factors [3]. Over 1990–2010, an annual average of 0.5 Gt carbon was released into the atmosphere due to deforestation [2]. Owing to these high forest emissions, the United Nations Framework Convention on Climate Change (UNFCCC) introduced Reducing Emissions from Deforestation and Degradation (REDD+) in 2007 [4], which was incorporated into the Paris Agreement in 2015 [5]. REDD+ aims to help developing countries reduce deforestation and forest degradation through various policy approaches and positive incentives, as well as forest conservation, sustainable forest management, and increasing forest carbon sink.

Accurate monitoring and measurement of emission reductions are especially important in the implementation of REDD+ projects. The potential benefits of REDD+ projects depend primarily on the precise measurement of the emission reductions [6], which can directly affect the stakeholder benefits obtained from REDD+ projects [7]. Thus, there is a need to assess the reliability of community-based monitoring and measurement of emission reductions, especially given the potential impact it may have on the cost-effectiveness and local acceptability of REDD+ projects [8]. The latter is particularly relevant to the situation of indigenous people (landholders), whose territory plays a vital role in above-ground carbon stocks in the tropics [9]. The legitimate participation of indigenous peoples in monitoring, reporting, and verification (MRV) is a fundamental principle that respects their human rights and will also promote their ownership of the REDD+ projects [10].

### The Problem and the Approach to Investigate

At present, reliable estimates of emission reductions remain very difficult. The high uncertainties in input data may seriously compromise the credibility of the estimates of emission reductions and jeopardize the feasibility of REDD+ as a mitigation option [11]. The existing studies examined the issue of uncertainty in estimates of emission reductions for REDD+, and mainly involved the uncertainty at the project scale [12], with the theoretical estimation of several error sources [13] or with the approach of dealing with uncertainty [14]. Existing research indicates that the uncertainty relates to measuring forest area, forest area changes and trends [12,15,16,17], forest carbon density [18,19,20], carbon emissions after deforestation and degradation [21,22], and the activities and processes contained in the accountability for land-use-change fluxes [23,24]. When uncertainty is equal to or greater than the promised emission reductions, compliance with REDD+ contracts will become particularly difficult to assess and may cause controversy [25]. Thus, it is critically important to evaluate the impacts of monitoring errors on the performance of REDD+ projects.

To reduce uncertainty in REDD+, a reliable MRV system for emission reductions is necessary. The cost to establish an MRV system is a transaction cost in REDD+ projects that includes investigation costs as well as computer equipment and software [26,27]. In addition to the transaction cost, there is another important type of costs in REDD+ projects, namely, opportunity costs resulting from the foregone revenues that deforestation would have generated for livelihoods and the national economy [28]. Only when income from the REDD+ projects is higher than opportunity costs will the emission reductions become the best choice for landholders [29]. The investors, as other essential stakeholders in REDD+ projects, can transform forest emission reductions into “forest carbon credits”. However, a reliable MRV system is required for investors to accurately estimate emission reductions, which would create transaction costs [26]. An important role of incentives is to cover the cost of emission reductions by selling “carbon credit” [30]. Thus, it is necessary to provide incentives for investors to make up the transaction costs of REDD+ programs [31]. Moreover, the uncertainty in estimates of emission reductions may make the carbon credits available to investors become overvalued, leading to an increase in the transaction costs of investors and ultimately impairing their interests [7]. Thus, when the acquired funds from the sale of “carbon credit” are sufficient to cover the transaction costs of emission reductions, an incentive for REDD+ investors will be created [30].

High transaction costs in REDD+ projects are not conducive to the implementation of REDD+ projects in developing countries. However, the transaction costs of REDD+ projects can be efficiently reduced if policymakers can provide an incentive for transaction costs [32]. Skutsch, Vickers, Georgiadou, and McCall [8] also found that the stakeholders with better negotiation skills and experience in tapping government incentives have more advantages in the implementation of REDD+ projects than the stakeholders with the lowest opportunity costs. People think that stakeholders should be given the incentive to improve the performance of REDD+ projects [33,34].

Multiple stakeholders are often involved in REDD+ projects, and the stakeholders’ benefits typically differ [35]. The general uncertainty is likely to have a direct impact on all stakeholders’ benefits, but which stakeholder has more advantages in uncertainty is still unclear. The overestimation and underestimation of emission reductions in REDD+ directly determine the number of carbon credits, which may affect the benefits of various stakeholders. Thus, high uncertainties around emission reduction estimates would have a significant impact on the interests of stakeholders [36]. Meanwhile, reducing uncertainty implies the need to take a series of measures, including the establishment of a more efficient MRV system, which may increase transaction costs in REDD+ projects [37]. It also means that more incentives are needed to provide enough incentive for stakeholders. Since forestry is the sector identified as having one of the largest uncertainties, it is necessary to consider uncertainty in the context of REDD+ incentives [38]. Our aim in the current study was to fill this research gap by examining how uncertainty and incentive can influence stakeholder benefits and emission reductions.

This paper focuses mainly on the effects of uncertainty and incentive on the performance of REDD+ projects, and makes the following three contributions: (i) illustrating the potential effects of uncertainty and incentive on emission reductions and stakeholder benefits of REDD+ projects through Stackelberg models and numerical simulations; (ii) demonstrating that the increase in the errors or incentives will lead to an increase in emission reductions and stakeholder profits, while the equilibrium errors and emission reductions are both related to the market size; and (iii) confirming that incentives for investors are better than incentives for landholders in reducing monitoring errors and improving the performance of REDD+ projects.

The structure of the paper is as follows: Section 2 briefly reviews the relevant literature on uncertainty, incentive, and their associations with the performance of REDD+ projects. Section 3 establishes several Stackelberg models to hypothetically examine the effects of uncertainty and incentive on the performance of REDD+ projects. Section 4 uses policy simulation to examine the relationship between uncertainty in estimates, incentive, and performance of REDD programs in different scenarios. Based on the above study, the discussion constitutes Section 5, followed by the conclusion in Section 6.

## 2. Literature Review

Existing literature on REDD+ has been very abundant, and mainly concerned with the international to the national level of the system design [39]. On summarizing the existing literature it can be seen that six topics dominate [40]. One is about the studies on performance-oriented financing in REDD+ [41,42,43]. The second topic is concerned with the institutional design and responsibilities in REDD+ [44,45,46]. The third topic is to examine the issues of uncertainties and MRV in REDD+ [47,48,49,50]. The fourth topic concerns the REDD+ safeguard, including land property rights [51,52,53], indigenous community participation [54,55,56], social safeguards and benefit sharing [57,58,59], and biodiversity conservation [60,61,62]. The fifth topic focuses on the issue of REDD+ reference levels [63,64,65]. The sixth topic examines the specific drivers of deforestation [66,67,68]. According to our research goals, this paper pays more attention to the relationship between uncertainty in estimates, incentive, and performance of REDD+ projects.

Past studies on uncertainties in the REDD+ projects demonstrated that the economic and political dimensions of REDD+ projects and their performance-based criteria require highly accurate monitoring, reporting, and verification of emissions from the forest [69]. The success of a REDD+ project depends on the ability of countries to provide measurable, reportable, and verifiable emission reductions [36]. Most studies focus on data availability in MRV systems [18,70,71,72] and related costs [73,74]. Some of the literature also finds that the current status of the MRV system is not satisfactory [40]. According to the REDD+ projects implemented in recent years, Joseph, Herold, Sunderlin, and Verchot [47] argue that MRV’s flaws are not only due to the limitation of technical expertise, but also to the slowness of international REDD+ policy development and the uncertainties in the development of the forest carbon market. Romijn, Herold, Kooistra, Murdiyarso, and Verchot [48] argue that the MRV capacity gaps in most developing countries are large, and monitoring capacity building needs to be part of the investment commitment in the performance-based REDD+ projects. However, none of the above literature focuses on the impacts of uncertainty caused by imperfect MRV on the performance of REDD+ projects.

So far only a few literature works involve the association of uncertainty with the performance of the REDD+ project. Pelletier, Ramankutty, and Potvin [36] argue that relevant uncertainties may overshadow the actual emission reductions in developing countries, and uncertainties arising from imperfect MRV may prevent widespread implementation of performance-based financing [75]. Bucki, et al. [76] as well as Pelletier, Martin, and Potvin [22] both argue that uncertainties in REDD+ projects may lead to the effects of changes in forest management remaining within error range for a few years, and thus REDD+ incentives cannot be allocated to the countries that actually reduce emissions based on a reliable benchmark. Lusiana, van Noordwijk, Johana, Galudra, Suyanto, and Cadisch [7] argue that the uncertainty of carbon emission estimates depends on scale, while local stakeholders’ views on reasonable REDD+ projects affect and limit transaction costs. Thus, uncertainty analysis can be used as the basis for determining an appropriate scale for monitoring carbon emission estimates, which can be used as a performance indicator for REDD+ incentives. This paper extends the above studies, but uses a Stackelberg game approach to examine the effects of uncertainty on stakeholder profitability in REDD+ projects.

Another core issue relating to the performance of REDD+ projects is the incentive scheme for investment, which encourages developing countries to conserve forests, and reduce deforestation and degradation [77]. There have been more studies on the incentive issue for public sector investors [78]. With the deepening of studies on incentives in REDD+ projects, some studies have focused on optimizing the market and stimulating global private investment to shift to mitigation and adaptation activities related to climate change [79]. However, Barron and McDermott [80] argue that an important reason to explain the lack of incentives for private investors is that a global REDD+ system of emissions trading or cap-and-trade has not yet formed. Carbon emission credits from REDD+ projects have been accepted by the California cap-and-trade program [43], but are excluded from the European Union Emission Trading Scheme (EU ETS) [81]. An important reason is that the EU insists that a transparent and accurate MRV system is urgently required for carbon emissions in the forest and agricultural sectors [82]. Since each member state has different reasons for deforestation and degradation, this may make it difficult for EU member states to agree on the effectiveness of REDD+. However, the EU reiterates the need to develop a policy to integrate land use and forestry into the 2030 Climate and Energy Policy Framework once the necessary technical conditions allow [82]. Thus, it indirectly indicates that REDD+ is likely to be incorporated in the EU ETS and even included in the global emissions trading system if the uncertainty issue can be effectively solved. Given that China launched the national emission trading system in 2017 [83], carbon credits from the REDD+ program and Clean Development Mechanism (CDM) are likely to be incorporated in the system in the future. The global emission trading market for REDD+ projects is currently under formation, and REDD+ projects still have great attraction for private investors due to the potentially large future market size [84].

Another critical issue related to the performance of REDD+ projects is the incentives for indigenous people. Pandey, et al. [85] argue that the motivation to realize REDD+ carbon incentives in indigenous community forests can contribute to the mitigation and adaptation of climate change. McDermott, et al. [86] argue that bilateral and multilateral financing initiatives have played a “funds-based” authority through the implementation of operational safeguards to protect indigenous and local communities, but limited funding and low capacity of REDD+ countries to absorb these funds have limited their impact. Thus, it is necessary to strengthen inclusive participation and improve incentive management within the indigenous communities if incentive-based conservation projects are to achieve their social development and environmental goals [87]. Similarly, Corbera, et al. [88] also reveal the role of incentives for indigenous peoples by examining governance and livelihood changes in the community-based participatory forest management in Tanzania. To effectively protect the rights of indigenous people, Savaresi [89] argues that concerns about the social impact of REDD+ projects can be addressed by establishing clearer and stronger links with human rights instruments. Moreover, Wright [90] argues that security tenure must be seen as a prerequisite for effective REDD+ that is sensitive to indigenous rights. Thus, the Paris Agreement in December 2015 regarded REDD+ as a critical policy tool for climate change mitigation and clearly recognized the need to respect human rights in all climate actions [57].

Although the values are diverse in REDD+, the neoliberal incentive remains a useful starting point for us to understand stakeholder behaviour in REDD+. We recognize that values are pluralistic in REDD+, including efficiency, equity, social and environmental justice, as well as poverty alleviation [91,92,93]. However, when we consider all the values in REDD+ design, the value pluralism may “risk reinforcing the problematic dimensions and entrenched power structures of the approach as a whole and hence work against the effective resolution of these very concerns” [94]. As Wunder [95] argued, the incentive in REDD+ should not be “burdened” with many public concerns around “alleviating poverty, gender, indigenous people, human rights, and other noble causes”, since this would limit private sector investment, resulting in REDD+ losing new financing options. Even in the experiment on power inequality in Nicaragua [96], the “payments for ecosystem services (PES) simulation game” is still based on the neoliberal incentives, which can increase our understanding of the complex negotiations between different participants involved in ecosystem service governance. Moreover, lots of theoretical and empirical studies have confirmed that stakeholders in REDD+ are driven by self-interest and economic rationality to obtain maximum profits from the forests they exploit and manage (e.g., Purnomo, et al. [97], Fosci [98], Fletcher and Breitling [99], Isenhour [100], and Boer [101]). Thus, REDD+ as an application of neoliberalism in forest conservation still relies on neoliberal incentives [99]. As REDD+ is incentive-driven, following the neoliberal logic, and often requires private contracts between landholders and investors rather than regulatory approaches, these are more effective and efficient than those policies that require governmental oversight [102].

The literature on the effects of incentives on the performance of REDD+ projects remains in its infancy. Busch, et al. [103] proposed an improved voluntary incentive structure for REDD+ projects by comparing the emission reductions under the mandatory incentive structure with the basic voluntary incentive structure. Boer [104] used the governmentality theory to explore the rationality of various incentives, arguing that REDD+ incentives are often combined with neoliberalism in climate agenda, that is, using the market instruments to achieve the commodification of forest carbon. However, who should get the incentive is still in debate. Palmer [105] believes that the investors could obtain REDD+ incentives in the form of tradable carbon credits without needing to transfer these rights on to individuals with forest resources. Sheng, Cao, Han, and Miao [31] argue that incentives for investors can benefit all landholders by comparing four incentive modes. On the contrary, Loaiza, et al. [106] argue that indigenous communities should receive more incentives, and the existing incentives are inadequate to encourage them to participate in REDD+ projects. Similarly, Salas, et al. [107] also tend towards providing more incentives to landholders by using self-enforcing contracts. Sheng and Qiu [108] identified a range of factors that influence incentives in REDD+ from the perspective of governmentality. Guadalupe, et al. [109] identified the primary drivers and agents of deforestation in high-forest and low-deforestation regions. Pandit [110] revealed the factors affecting respondent’s knowledge of REDD+ goal in Nepal. Albers, et al. [111] employed the lens of economic incentives to explore the underappreciated costs in REDD. However, the previous studies tend to ignore the role of uncertainty when analyzing incentives [25]. This paper extends the above analysis by considering both uncertainty and incentive within a theoretical framework, examining the REDD+ incentives as a techne of government that attempts to render forest conservation governable and administrable. The REDD+ incentives are regarded as a techne of government that is, “means, mechanisms, procedures, instruments, tactics, techniques, technologies, and vocabularies” that are used by governments to conduct individual conducts for combating environmental degradation [112]. The REDD+ incentives rely on mobilizing the techne of the government to achieve the spatial rationalization of emission reductions, especially concerning the REDD+ incentives, to consider the effects of uncertainty.

## 3. Methods

### 3.1. Incentive and Profit Functions

Incentive as a type of policy instrument, like a tax and other market-oriented tools, can internalize the social costs of REDD+ projects in accord with social benefits and private benefits [113]. Therefore, the possible incentive should be considered when analyzing stakeholders’ profit functions.

Before analyzing the effects of uncertainty and incentive on the performance of REDD+ projects, a scenario without an incentive should be considered first, namely the Business-As-Usual (BAU) scenario. The game players are REDD+ investors and landholders, and the landholders can gain benefits from REDD+ projects which are dependent on the emission reductions (*q*) and compensation payments per unit emission (*r*). Of course, this also means that the landholders should give up logging income and benefits from land use change (LUC) at the same time, namely opportunity costs (*c_o_*). The investors can obtain “forest carbon credits” by developing REDD+ projects. These credits can be sold in the market, so investors can gain benefits which are related to emission reductions (*q*) and the international market prices of carbon credits (*p*). The primary sources of the cost per forest carbon credit are compensation payments per unit emission (*r*) and transaction costs (*c_t_*). The transaction costs usually occupy a significant proportion of the total costs of the REDD+ projects [114].

Landholders are the compensation payment leaders, who can decide whether to participate in the REDD+ projects and determine the number of emission reductions. When opportunity costs are lower than the available benefits, landholders will supply emission reductions. Landholders as the compensation payment leaders in the Stackelberg model [31], therefore, will first decide the compensation payments (*r*) to maximize their benefits, while investors as the compensation payment followers can only determine the emission reductions (*q*) according to compensation payments (*r*).

Policymakers are assumed to provide a specific incentive (*s*) for stakeholders to reduce the transaction costs in REDD+ projects. Thus, the incentive will be no higher than the unit transaction cost per emission reductions (i.e., *s* ≤ *c_t_*). To get a definite conclusion, determining who should get the incentive, needs further analysis. The game players are assumed to be risk-neutral stakeholders, and there is completely symmetrical information in the model. The amount of carbon credits is the emission reductions (*q*), and the prices of carbon credits are *p*. We start with a Stackelberg model to keep the analysis simple and obtain sharper insights. According to Ferrer and Swaminathan [115], the demand function of carbon credits can be assumed as a linear and decreasing function of the prices of carbon credits *p* (i.e., *q* = *m* − *p*); where *m* > 0 is the size of the potential market for carbon credits.

### 3.2. Measuring Uncertainty

It is especially crucial that accurate monitoring and measurement of emission reductions are directly related to the actual benefits obtained by stakeholders from REDD+ projects. Through studying the uncertainties of REDD programs in Garner, Cameroon, Indonesia, Colombia, and Suriname, Plugge, Baldauf, and Köhl [6] find that the income from REDD programs depends on the magnitude of errors, while the impacts of transaction costs and the price of carbon are relatively small. Therefore, a reliable MRV system for carbon emissions is critical to ensure the effective implementation of the REDD+ project in the post-Kyoto era.

There is a higher uncertainty in the quantification process of carbon storage, and such errors would produce a far-reaching impact on the final financing incentive mechanism of REDD+ projects. Grassi, Monni, Federici, Achard, and Mollicone [11] argued that uncertainty and incompleteness should be taken into account in the quantification of emission reductions, and put forward corresponding conservative principles to solve the potential incompleteness and high uncertainty of estimation in REDD+ projects. Tanabe and Wagner [116] proposed that reliable minimum estimation (RME) should be adopted in assessing changes in soil carbon storage. There are significant differences between RME and a lower confidence interval: the confidence interval only takes into account the sampling errors, while the RME is aimed at the overall uncertainty, including sampling errors and other errors, such as prediction errors, measurement errors, and classification errors [117]. The RME can be used to measure the changes of carbon storage in REDD+ projects such that the RME is the difference between the lower error interval during the base period and the upper error interval during the reporting period. The confidence interval only considers sampling errors, so the magnitude of emission reductions obtained by RME is less than that calculated by the confidence interval. The monitoring error is assumed as *e* (0 ≤ *e* ≤ 1). The emission reductions provided by landholders are *q*, while the compensation payments paid for landholders are dependent on the RME value *q* (1 − *e*), due to the uncertainty. However, the actual emission reduction obtained by investors is *q*. The uncertainty ultimately leads to differences in incentives available to investors and landholders. Thus, the uncertainty is likely to change the income levels of the two players, and ultimately affects the performance of REDD+ projects. These effects are discussed in the following.

When uncertainty exists, the effects of incentive on stakeholders’ profits, and the profit difference between incentive objects, are also considered in this section.

### 3.3. Stakeholder Decision-Making Model in the Scenario without Incentive (BAU Scenario)

The profit functions of investors and landholders of REDD+ projects in the scenario without incentive (BAU scenario) are represented in the following equations respectively:

(1) $$\begin{array}{c} \max\;\pi_{I}^{BAU}=qp-q(1-e)r-q(1-e)c_{t}\\ \;\;\;\;\;\;\;\;=q(m-q)-q(1-e)r-q(1-e)c_{t} \end{array},$$

(2) $$\max\;\pi_{L}^{BAU}=q(1-e)r-q(1-e)c_{o},$$

s.t.   0≤e≤1.

Here *π_I_^BAU^* and *π_L_^BAU^* are the profits of investors and landholders respectively in the BAU scenario. To examine the effects of error (*e*) and incentive (*s*) on the performance of REDD+ projects, we assume that market size (*m*), error (*e*), incentive (*s*), opportunity cost (*c_o_*), and transaction cost (*c_t_*) are exogenous variables, and only carbon emission reductions (*q*) and compensation payment (*r*) are endogenous variables. According to Equations (1) and (2), the profit function of investors (*π_I_^BAU^*) is the concave function of carbon emission reductions (*q*), i.e., ∂^2^*π_I_^BAU^*/∂*q*^2^ < 0. It can ensure the adequacy of the first-order conditions and the uniqueness of the optimal solution. The landholders are compensation payment leaders in the Stackelberg model, so backward induction is used to solve the model. The investors’ best response function for landholders’ compensation payments can be obtained as follows by letting ∂*π_I_^BAU^*/∂*q* = 0:

(3) $$q=\frac{m-(1-e)r-(1-e)c_{t}}{2}.$$

Now, investors can decide the required emission reductions to maximize their profits according to the best response function. By inserting Equation (3) to Equation (2), and letting ∂*π_L_^BAU^*/∂*r* = 0, the optimal solution for the model can be obtained as follows:

(4) $$\{\begin{array}{l} r*=\frac{m+(1-e)(c_{o}-c_{t})}{2(1-e)}\\ q*=\frac{m-(1-e)(c_{o}+c_{t})}{4} \end{array}.$$

By inserting Equation (4) to Equations (1) and (2), the equilibrium profits of investors and landholders in the BAU scenario are as follows:

(5) $$\pi_{I}^{BAU}=\frac{{\left[m-(1-e)(c_{o}+c_{t})\right]}^{2}}{16},$$

(6) $$\pi_{L}^{BAU}=\frac{{\left[m-(1-e)(c_{o}+c_{t})\right]}^{2}}{8}.$$

By comparing Equations (5) and (6), we find that investors’ profits are half that of landholders’ in the BAU scenario. The main reason is that landholders as compensation payment leaders have the first-mover advantages so that they can obtain more profits than investors.

### 3.4. Stakeholder Decision-Making Model in Incentive Scenarios

#### 3.4.1. Incentive Scenario for Landholders (*s*_1_ Scenario)

Policymakers are assumed to provide an appropriate incentive (*s*_1_) for landholders to reduce the transaction costs of REDD+ projects. The policymakers will provide an incentive for landholders according to the size of emission reductions, and the incentive should be less than or equal to the transaction cost (i.e., *s*_1_ ≤ *c_t_*). Due to the uncertainty, the incentives are determined by the RME value of emission reductions *q* (1 − *e*). Consequently, investor and landholder profit functions are:

(7) $$\max\;\pi_{I}^{S_{1}}=q(m-q)-q(1-e)r-q(1-e)c_{t},$$

(8) $$\max\;\pi_{L}^{S_{1}}=q(1-e)r-q(1-e)c_{o}+q(1-e)s_{1},$$

s.t.   0≤e≤1.

According to the first order conditions of profit maximization (∂*π_I_^S^*^1^/∂*q* = 0), investors’ best response function is:

(9) $$q=\frac{m-(1-e)r-(1-e)c_{t}}{2}.$$

By inserting Equation (9) to Equation (8), and letting ∂*π_L_^S^*^1^/∂*r* = 0, the optimal solution for the model can be obtained as follows:

(10) $$\{\begin{array}{l} r*=\frac{m+(1-e)(c_{o}-c_{t}-s_{1})}{2(1-e)}\\ q*=\frac{m-(1-e)(c_{o}+c_{t}-s_{1})}{4} \end{array}.$$

The equilibrium profits of investors and landholders can be obtained respectively through the same method in the incentive scenario for landholders (*s*_1_ scenario):

(11) $$\pi_{I}^{S_{1}}=\frac{{\left[m-(1-e)(c_{o}+c_{t}-s_{1})\right]}^{2}}{16},$$

(12) $$\pi_{L}^{S_{1}}=\frac{{\left[m-(1-e)(c_{o}+c_{t}-s_{1})\right]}^{2}}{8}.$$

#### 3.4.2. Incentive Scenario for Investors (*s*_2_ Scenario)

In this scenario, to reduce the transaction costs of REDD+ projects, policymakers will provide an incentive (*s*_2_) to investors instead of landholders. The incentive should be less than or equal to the transaction cost (i.e., *s*_2_ ≤ *c_t_*). Investors and landholders’ profit functions are as follows:

(13) $$\max\;\pi_{I}^{S_{2}}=q(m-q)-q(1-e)r-q(1-e)c_{t}+qs_{2},$$

(14) $$\max\;\pi_{L}^{S_{2}}=q(1-e)r-q(1-e)c_{o},$$

s.t.   0≤e≤1.

The total incentives depend only on emission reductions *q*. Let ∂*π_I_^S^*^2^/∂*q* = 0, then investors’ best response function can be obtained:

(15) $$q=\frac{m+s_{2}-(1-e)r-(1-e)c_{t}}{2}.$$

Letting ∂*π_L_^S^*^2^/∂*r* = 0, the optimal solution is:

(16) $$\{\begin{array}{l} r*=\frac{m+s_{2}+(1-e)(c_{o}-c_{t})}{2(1-e)}\\ q*=\frac{m+s_{2}-(1-e)(c_{o}+c_{t})}{4} \end{array}.$$

Finally, in the incentive scenario for investors (*s*_2_ scenario) the equilibrium profits of investors and landholders are:

(17) $$\pi_{I}^{S_{2}}=\frac{{\left[m+s_{2}-(1-e)(c_{o}+c_{t})\right]}^{2}}{16},$$

(18) $$\pi_{L}^{S_{2}}=\frac{{\left[m+s_{2}-(1-e)(c_{o}+c_{t})\right]}^{2}}{8}.$$

## 4. Results

### 4.1. Numerical Specification

To further examine the impacts of uncertainty and incentive on the performance of REDD+ projects, numerical simulation is used to verify the conclusions obtained by theoretical models. Boucher [118] reviewed 29 papers on empirical studies on the opportunity costs in REDD+ projects, and found that the average opportunity cost was 2.51 USD/tCO_2_. Therefore, in this paper, the opportunity cost (*c_o_*) is set to 2.5 USD/tCO_2_. The generalized transaction can reach 1 USD/tCO_2_, which contains transaction costs of 0.38 USD/tCO_2_ [119], implementation costs of 0.58 USD/tCO_2_ [120] and administration costs of 0.04 USD/tCO_2_ [26]. Thus, 1 USD/tCO_2_ is selected as the transaction cost (*c_t_*).

First, we examine the effects of uncertainty on the performance of REDD+ projects, defining the error (*e*) as in the range of 0 ≤ *e* ≤ 10%. Meanwhile, because the primary purpose of the incentive is to reduce the transaction cost, the incentives *s*_1_ and *s*_2_ are set to the average value of 0.5 USD/tCO_2_. The market size (*m*) is set to 10. The other values are also used to test these parameters, and results show that the simulation results have strong robustness for parameter variations in the trends of emission reductions.

Second, we examine the impacts of incentive on the performance of REDD+ projects. Because the transaction cost (*c_t_*) is set to 1 USD/tCO_2_, the incentive (*s*_1_ and *s*_2_) ranges are between 0 USD/tCO_2_ and 1 USD/tCO_2_. The remaining parameters are set as follows: *e* = 5%, *m* = 10 to ensure the profit functions of both players are meaningful. The simulation results also have strong robustness for parameter variations in the trends of emission reductions after similar tests.

### 4.2. Numerical Simulation

**Figure 1:**
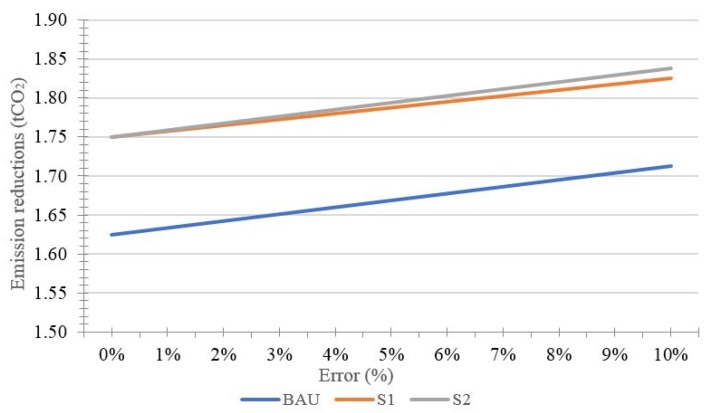
Effects of uncertainty on emission reductions.

**Figure 2:**
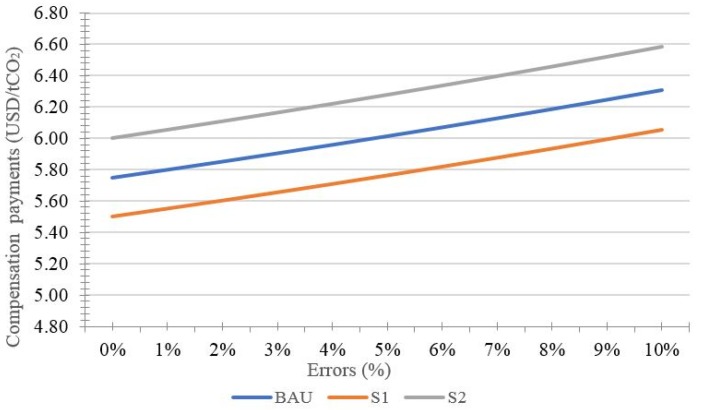
Effects of uncertainty on compensation payments.

By using the premise of invariable incentive, the effects of uncertainty on the performance of REDD+ projects are shown in Figure 1 and Figure 2, and Table 1.

According to Figure 1, the measured value of the emission reductions increased as errors increased. To maintain the previous profits, investors and landholders need to increase the emission reductions to make up for the underestimated amount caused by errors. Therefore, greater errors result finally in increased *r* measured value of emission reductions. It is worth noting that the emission reductions in the *s*_2_ scenario are greater than that in the *s*_1_ scenario, and this difference is more significant with an increase in errors. The emission reductions rise from 1.75 tCO_2_ (*e* = 0) to 1.83 tCO_2_ (*e* = 10%) in the *s*_1_ scenario, while the increase in emission reductions goes from 1.75 tCO_2_ (*e* = 0) to 1.84 tCO_2_ (*e* = 10%) in the *s*_2_ scenario. A conclusion, therefore, can be drawn that the effects of incentive for investors are better than those for landholders. Meanwhile, the greater the errors, the more significant the effects will be.

Due to the information asymmetry in the opportunity costs of emission reductions, investors are not aware of the real opportunity costs of landholders [121]. Thus, the landholders become the price leader and can first set the compensation payments for the emission reductions to maximize their profits [31]. According to Figure 2, it can be seen that the compensation payments also rise with the increase in errors. The reason may be that the underestimated emission reductions will become greater with rising errors. However, the actual emission reductions from the landholders are unchanged. Consequently, the landholders will ask for higher compensation payments to cover the costs of underestimated emission reductions. It is worth noting that the compensation payments in the *s*_1_ scenario are lower than that in the BAU scenario, while the compensation payments in the *s*_2_ scenario are higher than that in the BAU scenario. It suggests that incentive for landholders will reduce compensation payments, while incentive for investors will increase compensation payments.

The influence of errors on the profits of REDD+ stakeholders is shown in Table 1. It can be seen that the profits of landholders and investors will have a corresponding increase when errors arise. Rising errors not only increase the actual emission reductions, but also lead to the compensation payments rising. Even though the emission reductions may be underestimated due to errors, stakeholder profits can still increase. However, the increased magnitude is different in different scenarios. When errors increase from 0 to 10%, stakeholder profits are increased by 8.76% and 10.25% in the *s*_1_ and *s*_2_ scenarios respectively. It suggests that incentives for investors are more likely to promote an increase in stakeholder profits.

**Figure 3:**
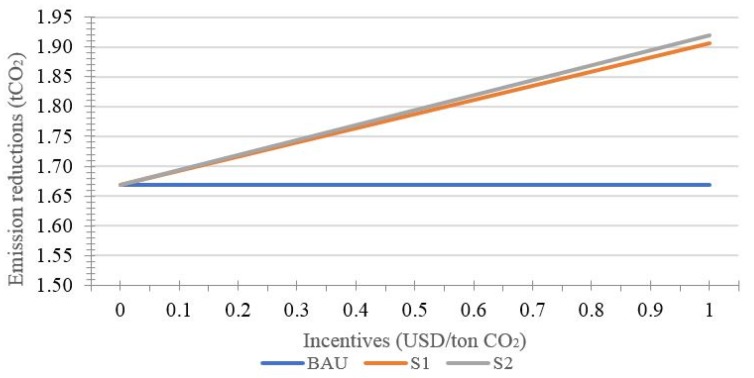
Effects of incentive on emission reductions.

**Figure 4:**
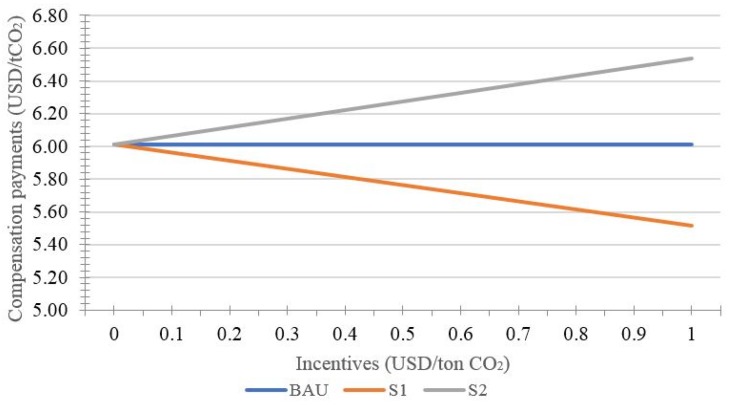
Effects of incentive on compensation payments.

The following Figure 3 and Figure 4 and Table 2 show the effects of incentive on the performance of REDD+ projects when the monitoring error is constant.

According to Figure 3, the emission reductions rise with increased incentive in the two incentive scenarios when the error is unchanged. Through further comparison of the two kinds of incentive modes, it can be found that incentive for investors will cause emission reductions to increase more significantly. When the incentive rises from 0 USD to 1 USD, the emission reductions rise from 1.67 tCO_2_ to 1.91 tCO_2_ in the *s*_1_ scenario, while increasing from 1.67 tCO_2_ to 1.92 tCO_2_ in the *s*_2_ scenario. It once again shows that the effects of incentive for investors are better than that of incentive for landholders. Moreover, the difference between the two scenarios is more significant with rising incentive.

Furthermore, the effects of the two incentive modes on compensation payments move in the opposite direction, as shown in Figure 4. The incentive for landholders will sharply decrease the compensation payments, and the more incentives are provided, the lower the compensation payments. On the other hand, the incentive for investors can significantly increase the compensation payments.

The impacts of incentive on the profits of REDD+ stakeholders are shown in Table 2. Comparing stakeholder profits in the three scenarios, we find that incentive can significantly increase the profits of investors and landholders, but the effects of incentive for investors would be better. The stakeholder profits are increased by 30.49% and 32.21% in the *s*_1_ and *s*_2_ scenarios respectively, when incentives are increased from 0 USD to 1 USD. It is again proven that the effects of incentive for investors have better effects than the effects of incentive for landholders.

## 5. Discussion

### 5.1. The Scenarios with Endogenous Errors

In the previous analysis, the market size (*m*), error (*e*), incentive (*s*), opportunity cost (*c_o_*), and transaction cost (*c_t_*) are assumed to be exogenous variables, and carbon emission reductions (*q*) and compensation payment per carbon emission reductions (*r*) are assumed to be endogenous variables. Because landholders are the compensation payment leaders in the Stackelberg models, the equilibrium compensation payments and emission reductions can be obtained by solving the models. To examine the impact factors of errors, we assume that *m*, *r*, *s*, *c_o,_* and *c_t_* are exogenous variables, while *q* and *e* are endogenous variables. Similarly, landholders as the leaders can choose the appropriate error levels for maximizing their profits, while investors will determine the equilibrium emission reductions according to the given errors. The investors’ best response function for landholders’ monitoring errors can be obtained through the same method in the scenario without incentive (BAU scenario):

(19) $$q=\frac{m-(1-e)r-(1-e)c_{t}}{2}.$$

Now investors can decide the required emission reductions that will maximize their profits according to the best response function. Consequently, the equilibrium solution is as follows:

(20) $$\{\begin{array}{l} e*=1-\frac{m}{2(r+c_{t})}\\ q*=\frac{m}{4} \end{array}.$$

The corresponding profits of investors and landholders in the BAU scenario are as follows:

(21) $$\pi_{I}^{BAU}=\frac{m^{2}}{16},$$

(22) $$\pi_{L}^{BAU}=\frac{m^{2}(r-c_{o})}{8(r+c_{t})}.$$

Similarly, we can analyze the *s*_1_ scenario. The investors’ best response function is:

(23) $$q=\frac{m-(1-e)r-(1-e)c_{t}}{2}.$$

The equilibrium errors (*e*) and emission reductions (*q*) in the *s*_1_ scenario can be obtained by using the same method:

(24) $$\{\begin{array}{l} e*=1-\frac{m}{2(r+c_{t})}\\ q*=\frac{m}{4} \end{array}.$$

The profits of investors and landholders in the *s*_1_ scenario are:

(25) $$\pi_{I}^{BAU}=\frac{m^{2}}{16},$$

(26) $$\pi_{L}^{BAU}=\frac{m^{2}(r\;-\;c_{o}+s_{1})}{8(r+c_{t})}.$$

Accordingly, the investors’ best response function in the *s*_2_ scenario is as follows:

(27) $$q=\frac{m+s_{2}-(1-e)r-(1-e)c_{t}}{2}.$$

Now, the equilibrium *e* and *q* in the *s*_2_ scenario are:

(28) $$\{\begin{array}{l} e*=1-\frac{m+s_{2}}{2(r+c_{t})}\\ q*=\frac{m+s_{2}}{4} \end{array}.$$

The corresponding profits of investors and landholders in the *s*_2_ scenario are as follows:

(29) $$\pi_{I}^{BAU}=\frac{{\left(m+s_{2}\right)}^{2}}{16},$$

(30) $$\pi_{L}^{BAU}=\frac{{\left(m+s_{2}\right)}^{2}(r\;-\;c_{o})}{8(r+c_{t})},$$

### 5.2. Scenario Comparison

When the uncertainty factor of REDD+ projects is no longer exogenous, the impact factors of errors can be further examined. According to Equation (24), the equilibrium error (*e*) in the BAU scenario is determined by three variables: error (*e*) is inversely proportional to the market size (*m*), and proportional to the compensation payment (*r*) and the transaction cost (*c_t_*). It means that the scale effect will gradually reduce the marginal transaction costs of REDD+ projects, and the MRV system will be perfect for the expansion of market size. Consequently, the errors become smaller than before. Similarly, an increase in compensation payment (*r*) and transaction cost (*c_t_*) may result in the reduction of market size (*m*), eventually causing error (*e*) to increase. The most interesting phenomenon is that the equilibrium carbon emission reductions (*q*) have nothing to do with any variables except for the market size (*m*). This indicates that the equilibrium carbon emission reductions will increase with the expansion of market size. Now the incentive scenarios are discussed through a scenario comparison, with specific results shown in Table 3.

According to the comparison in Table 3, it can be found that the equilibrium error and carbon emission reductions in the incentive scenario for landholders (*s*_1_ scenario) are the same as those in the BAU scenario. It suggests that incentive for landholders cannot decrease the error or increase carbon emission reductions. Compared with the BAU scenario, the error will decline, and the carbon emission reductions will increase with the increase in the incentive for investors (*s*_2_). It suggests that incentive for investors can improve the performance of REDD+ projects.

The following examines the profit changes of investors and landholders. According to Equations (21) and (22), investor profits are only related to the market size, while landholder profits are decided by market size (*m*), compensation payment (*r*), opportunity cost (*c_o_*), and transaction cost (*c_t_*) in the BAU scenario. The stakeholder profits in the three scenarios are compared in Table 4.

When policymakers provide an incentive for landholders, landholder profits will increase with the increase in incentive (*s*_1_), while investor profits will not change. However, the incentive for investors will make profits for both landholders and investors increase. Also, the added value of profits will be higher than the incentive (*s*_2_), due to the quadratic term in Equations (29) and (30). The results are similar to the findings of Sheng, Cao, Han, and Miao [31]. However, the effects of uncertainty on the emission reductions and stakeholders’ benefits have not been captured in the latter. In fact, the above results confirmed that a rise in uncertainty indeed leads to an increase in the emission reductions and stakeholders’ benefits. Thus, it is important to integrate incentives to take uncertainty into account in designing an REDD+ scheme.

## 6. Conclusions

Sustainable development has become a critical issue in the political agenda across different countries [122,123,124,125]. It is a new measure of the international community’s resolve to mitigate climate change in that the REDD+ scheme provides an incentive to developing countries to eliminate the historical tendency to deforest as part of the development process. The universal existence of uncertainty in REDD+ projects, however, may have impacts on stakeholder benefits. Therefore, the MRV system for carbon emission reductions is key to the successful implementation of REDD+ projects. Of course, the establishment of the MRV system is not without financial cost, and high transaction costs are not conducive to the implementation of REDD+ projects. Thus, providing an incentive for transaction costs is a valuable policy tool to promote the effective implementation of REDD+ projects. The factors of uncertainty in REDD+ projects were further discussed in this paper, using Stackelberg models and simulation research to examine the effects of uncertainty and incentive on performance and stakeholder benefits. Finally, we compared the equilibrium errors, emission reductions, and stakeholder benefits in different scenarios. The results show that:

1. When the incentive is constant, the emission reductions and compensation payments as endogenous variables will increase as errors increase. Eventually, the profits of various stakeholders will also simultaneously increase.
2. When errors are unchanged, the emission reductions and stakeholder profits will increase with rising incentive. Meanwhile, the effects of incentive for investors will be better than those for landholders, and the difference is more significant with increased incentive and errors.
3. When the emission reductions and errors are endogenous variables, the monitoring error is inversely proportional to the market size, and proportional to the compensation payment and transaction cost. Also, the equilibrium emission reductions are related only to market size.
4. The incentive for landholders cannot improve the performance of REDD+ projects, but only increase landholder profits. However, the incentive for investors can reduce error, increase emission reductions, and improve the welfare of all stakeholders.

The above results have some implications for policy-making: (i) to improve the performance of REDD+ projects, policymakers could consider using the provision of incentive for investors in REDD+ projects to reduce carbon emissions; (ii) the market size of REDD+ projects should be expanded as much as possible, thereby reducing the marginal transaction costs; reduction in monitoring errors is conducive to the establishment of a reliable MRV system; (iii) it is worth noting that the transaction costs of REDD+ projects should be reduced as much as possible. Previous analysis has proven that a decrease in transaction costs can contribute to the market expansion of REDD+ projects, eventually decreasing monitoring errors.

Since incentive for the stakeholders, in case of high uncertainties, could result in wasted financial resources and abuses of the payment scheme itself, it is necessary to provide incentives based on performance to developing countries to establish effective and credible MRV systems. Thus, the performance-based international payment incentives (such as the widely discussed Carbon Fund of the Forest Carbon Partnership Facility (FCPF)) have the potential to reduce uncertainties and provide more effective incentives for developing countries to boost their MRV systems in the future when assessing REDD+ performance [126].

REDD+ projects can not only bring benefits for investors and landholders, but also improve the environmental and ecological welfare of the whole society. External interests, however, have not been fully reflected in the price of “carbon credits.” Thus, future research needs to pay more attention to ensure that distribution is as fair as possible and not allow these incentives to be captured disproportionately by the most powerful stakeholders.

## Figures and Tables

**Table 1:** Effects of uncertainty on stakeholder profits in Reducing Emissions from Deforestation and Degradation (REDD+) projects (USD). BAU = Business-As-Usual.

Stakeholders	Scenarios	Error (%)										
		0	1%	2%	3%	4%	5%	6%	7%	8%	9%	10%
Investors	BAU	2.64	2.67	2.70	2.73	2.76	2.78	2.81	2.84	2.87	2.90	2.93
	*s*_1_	3.06	3.09	3.12	3.14	3.17	3.20	3.22	3.25	3.28	3.30	3.33
	*s*_2_	3.06	3.09	3.12	3.16	3.19	3.22	3.25	3.28	3.31	3.34	3.38
Landholders	BAU	5.28	5.34	5.40	5.45	5.51	5.57	5.63	5.69	5.75	5.81	5.87
	*s*_1_	6.13	6.18	6.23	6.28	6.34	6.39	6.44	6.50	6.55	6.61	6.66
	*s*_2_	6.13	6.19	6.25	6.31	6.37	6.44	6.50	6.56	6.62	6.69	6.75

**Table 2:** Effects of incentive on stakeholder profits in REDD+ projects (USD).

Stakeholders	Scenarios	Incentive (USD/tCO_2_)										
		0.00	0.10	0.20	0.30	0.40	0.50	0.60	0.70	0.80	0.90	1.00
Investors	BAU	2.78	2.78	2.78	2.78	2.78	2.78	2.78	2.78	2.78	2.78	2.78
	*s*_1_	2.78	2.86	2.95	3.03	3.11	3.20	3.28	3.37	3.45	3.54	3.63
	*s*_2_	2.78	2.87	2.95	3.04	3.13	3.22	3.31	3.40	3.49	3.59	3.68
Landholders	BAU	5.57	5.57	5.57	5.57	5.57	5.57	5.57	5.57	5.57	5.57	5.57
	*s*_1_	5.57	5.73	5.89	6.06	6.22	6.39	6.56	6.73	6.91	7.09	7.27
	*s*_2_	5.57	5.74	5.91	6.08	6.26	6.44	6.62	6.80	6.98	7.17	7.36

**Table 3:** Equilibrium errors and emission reductions in different scenarios.

Equilibrium Solution	Scenario without Incentive (BAU Scenario)	Incentive Scenarios	
		Incentive for Landholders (*s*_1_ Scenario)	Incentive for Investors (*s*_2_ Scenario)
Monitoring error *e*	1−*m*/[2(*r* + *c_t_*)]	1−*m*/[2(*r* + *c_t_*)]	1− (*m* + *s*_2_)/[2(*r* + *c_t_*)]
Emission reductions *q*	*m*/4	*m*/4	(*m* + *s*_2_)/4

**Table 4:** Stakeholder profits in different scenarios.

Players	Scenario without Incentive (BAU Scenario)	Incentive Scenarios	
		Incentive for Landholders (*s*_1_ Scenario)	Incentive for Investors (*s*_2_ Scenario)
Investors	*m*^2^/16	*m*^2^/16	(*m* + *s*_2_)^2^/16
Landholders	*m*^2^(*r* − *c_o_*)/[8(*r* + *c_t_*)]	*m*^2^(*r*−*c_o_* + *s*_1_)/[8(*r* + *c_t_*)]	(*m* + *s*_2_)^2^(*r*−*c_o_*)/[8(*r* + *c_t_*)]
